# Supplementary material for: l-Proline Alleviates Kidney Injury Caused by AFB1 and AFM1 through Regulating Excessive Apoptosis of Kidney Cells
Source: Toxins (Basel). 2019 Apr 16;11(4):226. doi: 10.3390/toxins11040226 (PMC6521284; doi:10.3390/toxins11040226)
Supplement: Supplementary file 1 [file toxins-11-00226-s001.pdf]

*Toxins* **2019**, *11*, 226; doi:10.3390/toxins11040226 www.mdpi.com/journal/toxins 

# Supplementary Materials: L-Proline Alleviates Kidney Injury Caused by AFB1 and AFM1 through Regulating Excessive Apoptosis of Kidney Cells

**Huiying Li †, Songli Li †, Huaigu Yang, Yizhen Wang, Jiaqi Wang and Nan Zheng ***

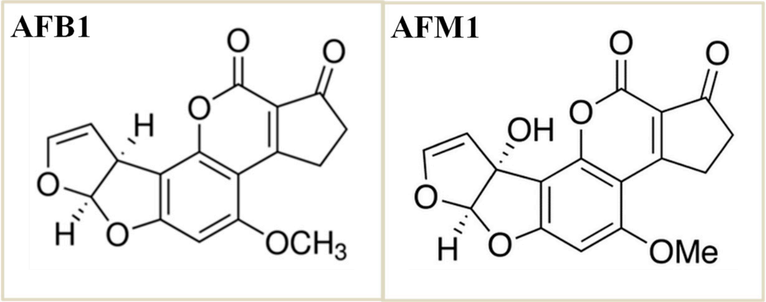


**Figure S1.** Chemical structure of aflatoxin B1 (AFB1) (**A**) and aflatoxin M1 (AFM1) (**B**).

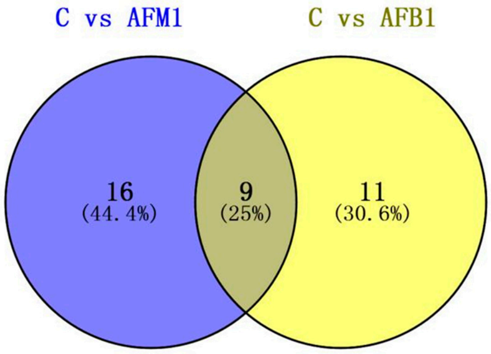


**Figure S2.** Special metabolites overlapped in different groups (control, AFB1 group and AFM1 group) in mice kidneys (VENN plot), through metabonomics detection and data analysis.
